# Changes in multimodal MRI parameters following orbital decompression surgery for thyroid-associated ophthalmopathy and their associations with clinical outcomes

**DOI:** 10.3389/fmed.2026.1870430

**Published:** 2026-08-17

**Authors:** Mingyue Zheng, Huan Zhao, Yin Liu, Wei Xiong

**Affiliations:** 1 Department of Ophthalmology, The Third Xiangya Hospital, Central South University, Changsha, Hunan, China; 2 Department of Radiology, The Third Xiangya Hospital, Central South University, Changsha, Hunan, China

**Keywords:** clinical outcomes, multimodal magnetic resonance imaging, orbital decompression, thyroid-associated ophthalmopathy, visual function assessment

## Abstract

**Objective:**

To evaluate postoperative changes in multimodal quantitative magnetic resonance imaging (MRI) parameters after orbital decompression in thyroid-associated ophthalmopathy and explore their associations with clinical outcomes.

**Methods:**

This retrospective study included 47 patients (61 eyes) assessed before and 6 months after surgery. Eye-level longitudinal outcomes were analyzed using linear mixed-effects models with random intercepts for patients and eyes nested within patients. Graves’ Ophthalmopathy Quality of Life questionnaire (GO-QOL) outcomes were analyzed using patient-level mixed-effects models. MRI–clinical associations were assessed using generalized estimating equations or multivariable linear regression. Benjamini–Hochberg false discovery rate correction was applied within prespecified hypothesis families.

**Results:**

After correction, proptosis and best-corrected visual acuity improved significantly, as did visual-function, appearance, and total GO-QOL scores. Multiple structural and diffusion-related MRI parameters also changed significantly after surgery. Greater reductions in corneal protrusion distance were associated with greater reductions in proptosis (*q* = 0.008). At the patient level, greater reductions in the vertical orbital apex area index and fat space apparent diffusion coefficient were associated with greater improvements in visual-function and total GO-QOL scores. No MRI parameter remained associated with appearance improvement after correction. A significant time-by-disease-subtype interaction was observed only for best-corrected visual acuity.

**Conclusion:**

Multimodal quantitative MRI detected postoperative structural and diffusion-related changes after orbital decompression. Reductions in the vertical orbital apex area index and fat space apparent diffusion coefficient showed exploratory associations with patient-reported visual-function improvement, although robustness varied across sensitivity analyses. These measures may complement, but not replace, clinical assessment.

## 1 Introduction

Thyroid-associated ophthalmopathy (TAO), also known as thyroid eye disease, is an autoimmune disorder closely related to Graves’ disease, with clinical manifestations including periorbital edema, exophthalmos, and diplopia, and in a few cases, optic nerve compression (1). Orbital decompression surgery is a core treatment for moderate to severe TAO and dysthyroid optic neuropathy (DON) (2), effectively expanding the orbital cavity, reducing the pressure at the orbital apex, and alleviating mechanical compression on the optic nerve (3).

Currently, clinical efficacy assessment mainly relies on visual acuity, visual field defects, intraocular pressure, and other ophthalmic examinations (4). Optical coherence tomography is another accessible and cost-effective tool for assessing retinal nerve fiber layer and ganglion cell complex changes in TAO (5). However, these assessments cannot directly evaluate retrobulbar extraocular muscles, orbital soft tissues, orbital apex crowding, or subtle postoperative intraorbital structural changes (6).

Magnetic resonance imaging (MRI) provides high soft-tissue contrast and multiplanar imaging, making it useful for evaluating orbital involvement in TAO (7). Multiple imaging studies have shown that MRI can help assess extraocular muscles, orbital soft tissue, and orbital apex crowding, also providing information on disease stage and tissue characteristics (8). Among MRI sequences, T2-weighted imaging, diffusion-weighted imaging (DWI) (9), and diffusion tensor imaging (DTI) have been used to evaluate orbital anatomy and diffusion-related tissue changes (10). Zhang et al. reported that the optic nerve diameter and optic nerve sheath diameter at the orbital apex were lower in patients with TAO than in healthy controls (11). Hu et al. found that the orbital apex muscle index (MI), optic nerve area index (ONAI), and optic nerve signal intensity ratio (SIR) were higher in patients with DON than in those with TAO (12). Liu et al. reported lower fractional anisotropy (FA) of the optic nerve in DON patients than in TAO patients (13).

However, current studies have mainly focused on the use of MRI for preoperative diagnosis and disease grading in TAO, while postoperative multimodal MRI changes after orbital decompression remain less well described. Therefore, this study utilized multimodal quantitative MRI to evaluate postoperative changes following orbital decompression in patients with TAO, and to explore the relationship between these changes and clinical outcomes. The goal was to provide imaging data that could complement routine clinical evaluations during postoperative follow-up.

## 2 Materials and methods

### 2.1 Patient characteristics

This retrospective study was conducted in accordance with the Declaration of Helsinki and was approved by the Ethics Review Committee of the Third Xiangya Hospital (Approval No. fast 25570). The requirement for informed consent was waived by the ethics committee. Information of patients who underwent orbital decompression surgery from October 2024 to November 2025 was collected. After screening based on the following criteria, a total of 47 patients (61 eyes) were finally included in the analysis.

Inclusion criteria: (1) diagnosed with TAO according to the diagnostic criteria of the European Group on Graves’ Orbitopathy (EUGOGO) (14); (2) no treatment with glucocorticoids, immunosuppressants, or orbital radiotherapy within the preceding 3 months; (3) normal thyroid function during the preceding 3 months.

Exclusion criteria: (1) amblyopia, cataract, pre-existing strabismus unrelated to TAO, or a history of other ophthalmic surgery; (2) proptosis or orbital swelling caused by other orbital diseases, including orbital tumors, idiopathic orbital inflammation, or orbital cellulitis; (3) inability to complete MRI examinations. TAO-related strabismus was not an exclusion criterion; these eyes were retained, and strabismus status was included as an eye-level covariate in the longitudinal and association analyses.

### 2.2 Data collection

Demographic data, best-corrected visual acuity (BCVA), intraocular pressure (IOP), proptosis degree, and strabismus were collected. Visual field index (VFI), mean deviation (MD), pattern standard deviation (PSD), and visual evoked potential measurements, including P100 latency and amplitude, were also recorded (15). Clinical activity score (CAS) and NOSPECS classification were evaluated. Each patient completed the Graves’ Ophthalmopathy Quality of Life questionnaire (GO-QOL), including visual function and appearance scores. Thyroid-related laboratory tests, including thyroglobulin (TG), thyroglobulin antibody (TgAb), thyroid peroxidase antibody (TPOAb), thyrotropin receptor antibody (TRAb), free triiodothyronine (FT3), free thyroxine (FT4), and thyroid stimulating hormone (TSH), were performed within 3 days before surgery. Preoperative orbital MRI was performed after admission. Decimal BCVA values were converted to logMAR before statistical analysis.

### 2.3 MRI scanning and image analysis

Magnetic resonance imaging was performed using a 3.0-T scanner (Ingenia Elition X; Philips Healthcare) with a 32-channel head coil. Participants were examined in the supine position with the head fixed by foam pads. They were instructed to keep their eyes closed, remain still, and stay awake during scanning. The protocol included axial, coronal, and sagittal fat-suppressed T2-weighted imaging (FS-T2WI), axial diffusion tensor imaging (DTI), and coronal diffusion-weighted imaging (DWI). Detailed acquisition parameters are provided in Supplementary Table 1. DTI images and tensor maps were generated using the standard processing pipeline of the Philips IntelliSpace Portal workstation, without additional offline preprocessing.

Radiologist 1 performed all MRI measurements used in the primary analyses. Both radiologists were blinded to the clinical subgroup and imaging time point. For reliability assessment, Radiologist 1 repeated the measurements in a randomly selected subset of 10 eyes after a 2-week interval, and Radiologist 2 independently evaluated the same 10 eyes. The initial measurements obtained by Radiologist 1 were used for the primary analyses. Detailed intraclass correlation coefficient (ICC) methods and results are provided in Supplementary Table 2, and Bland–Altman plots for representative parameters are shown in Supplementary Figure 1.

**FIGURE 1:**
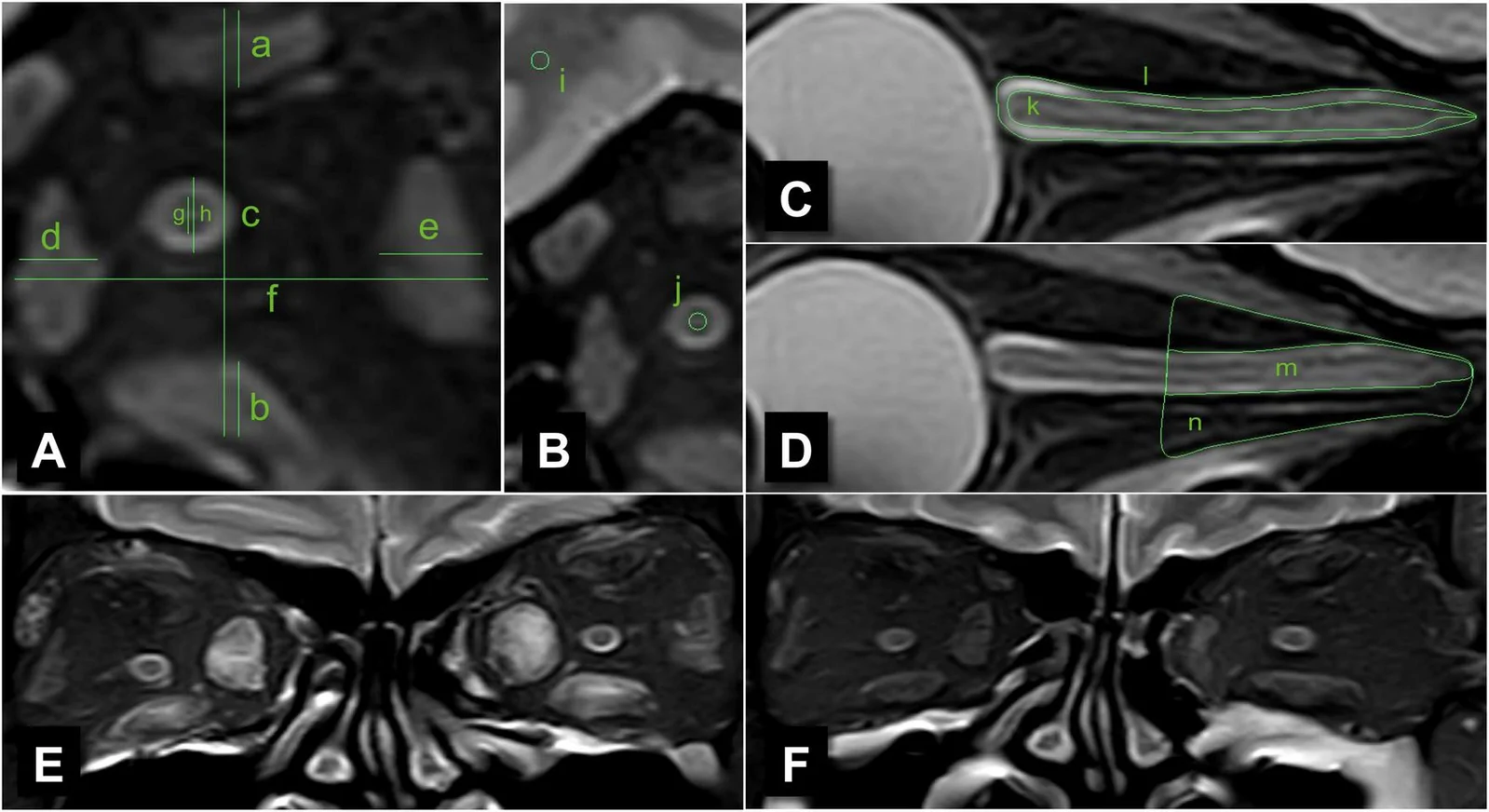
Orbital measurement and pre- and postoperative comparison. **(A,B)** Fifth retrobulbar coronal FS-T2WI images for VMI, HMI, ONDI, ONAI-C, and SIR measurements; VMI = (a + b)/c, HMI = (d + e)/f, ONDI = g/h, and SIR = SI_*j*_/SI_*i*_. **(C,D)** Sagittal FS-T2WI images for ONAI-S and OAAI-V measurements; ONAI-S = k/l, OAAI-V = m/n, with ONAI-A and OAAI-H measured similarly on axial images. **(E,F)** Preoperative and 6-month postoperative MRI in a 36-year-old male patient after orbital decompression, demonstrating marked resolution of extraocular muscle hypertrophy and orbital apex crowding. FS-T2WI, fat-suppressed T2-weighted imaging; VMI, vertical muscle index; HMI, horizontal muscle index; ONDI, optic nerve diameter index; SIR, signal intensity ratio; ONAI, optic nerve area index (coronal/axial/sagittal); OAAI, orbital apex area index (vertical/horizontal).

According to previous studies (12, 16, 17), the horizontal muscle index (HMI), vertical muscle index (VMI), optic nerve diameter index (ONDI), coronal optic nerve area index (ONAI-C), and signal intensity ratio (SIR) were measured on the fifth retrobulbar coronal FS-T2WI slice (Figures 1A, B).

**FIGURE 2:**
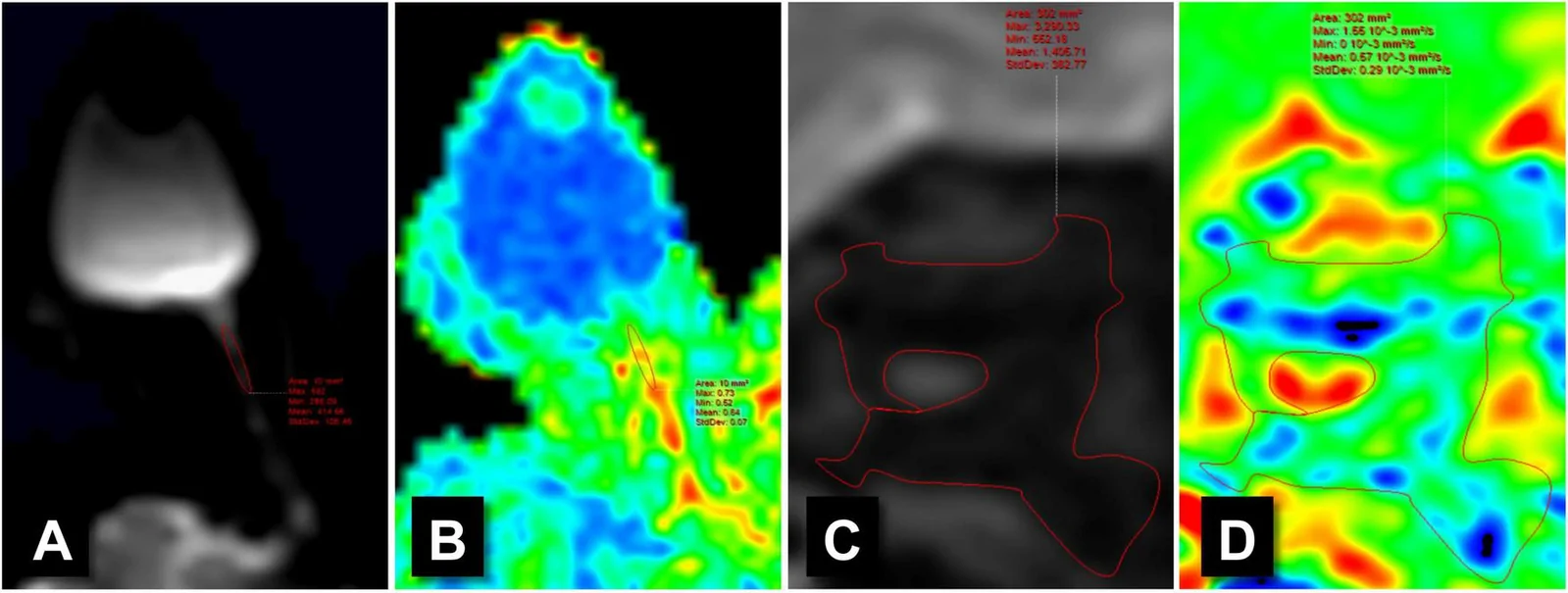
Measurement of orbital diffusion-related MRI parameters. **(A,B)** A 10 mm^2^ ROI was placed on the optic nerve on axial DTI images **(A)** and mapped to color-coded FA maps **(B)** for FA quantification; **(C,D)** ROIs of the intraconal fat space were drawn on DWI images **(C)**, and ADC values were obtained on corresponding ADC maps **(D)**. ADC values of the optic nerve and extraocular muscles were measured similarly. ROI, region of interest; DTI, diffusion tensor imaging; FA, fractional anisotropy; DWI, diffusion-weighted imaging; ADC, apparent diffusion coefficient.

The axial optic nerve area index (ONAI-A), sagittal optic nerve area index (ONAI-S), horizontal orbital apex area index (OAAI-H), vertical orbital apex area index (OAAI-V), and corneal protrusion distance (COD) were measured on axial or sagittal FS-T2WI images (Figures 1C, D). Representative preoperative and 6-month postoperative FS-T2WI images demonstrating resolution of extraocular muscle hypertrophy and orbital apex crowding are shown in Figures 1E, F. FA was measured using a 10-mm^2^ region of interest (ROI) placed on the optic nerve on axial DTI-FA maps (Figures 2A, B). Mean apparent diffusion coefficient (ADC) values of the optic nerve, extraocular muscles, and orbital fat were obtained from ROIs placed on the third to fifth retrobulbar slices of coronal DWI-ADC maps (Figures 2C, D).

### 2.4 Orbital decompression surgery

**FIGURE 3:**
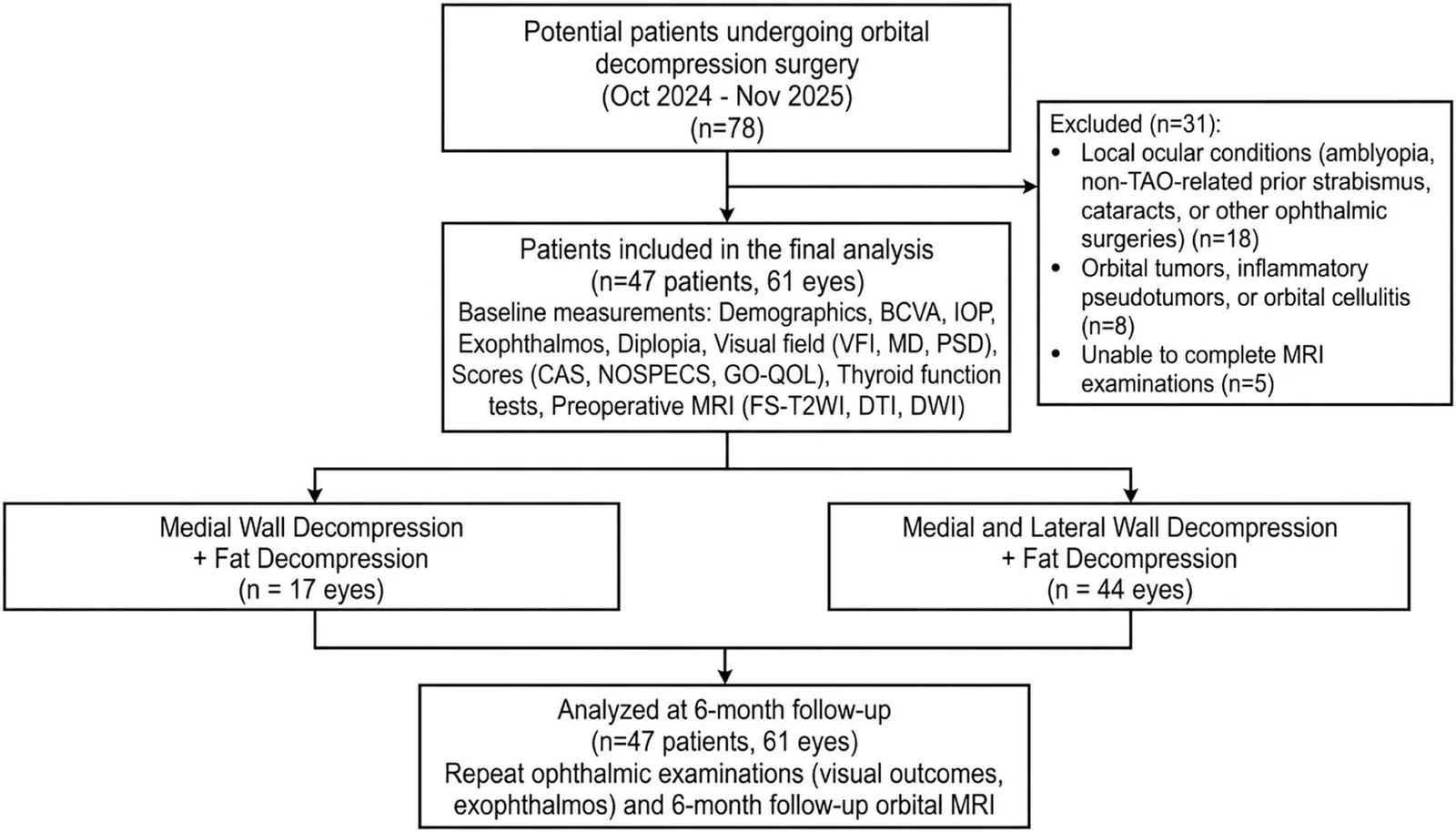
Workflow. Flow diagram of patient screening, surgical procedures, and 6-month follow-up. BCVA, best-corrected visual acuity; IOP, intraocular pressure; VFI, visual field index; MD, mean deviation; PSD, pattern standard deviation; CAS, clinical activity score; GO-QOL, Graves’ Ophthalmopathy Quality of Life questionnaire; FS-T2WI, fat-suppressed T2-weighted imaging; DTI, diffusion tensor imaging; DWI, diffusion-weighted imaging.

All surgeries were performed by the same experienced professor specializing in oculoplastic and orbital diseases (X.W., with 20 years of orbital surgery experience). The decompression method was selected according to surgical indications and patient preference (18, 19). Seventeen eyes underwent medial wall decompression combined with fat decompression and were classified as lateral wall not included. Forty-four eyes underwent medial and lateral wall decompression combined with fat decompression and were classified as lateral wall included. Postoperatively, all patients received intravenous methylprednisolone at 500 mg daily and broad-spectrum antibiotics for 3 days. Follow-up visits were scheduled at 1 week and 1, 3, and 6 months after surgery. The final ophthalmic examination results were recorded at 6 months postoperatively, and orbital MRI was repeated at the 6-month follow-up. The workflow is illustrated in Figure 3.

### 2.5 Statistical analysis

All analyses were performed using R version 4.5.2. Patient-level variables were analyzed in 47 patients, whereas eye-level variables were analyzed in 61 eyes. Continuous variables are presented as mean ± standard deviation or median [interquartile range], as appropriate, and categorical variables as n (%).

Longitudinal eye-level outcomes were analyzed using linear mixed-effects models fitted by restricted maximum likelihood with lmerTest::lmer(). The primary model was specified as outcome ∼ time + age + sex + group + period + SurgType + strabismus + NOS PECS + (1 | patientid/eyeid), with eyes nested within patients. Adjusted preoperative and postoperative estimates and postoperative changes with 95% confidence intervals were obtained from the fitted models. All 25 primary models converged; eight were classified as singular because one or more random-effect variance components were estimated at or near zero. Covariance-structure sensitivity analyses were performed using nlme::lme(). For PSD, an unstructured within-eye correlation structure with time-specific residual variances was used. Detailed model specifications and diagnostics are provided in the Supplementary Statistical Methods and Supplementary Table 5.

Patient-level GO-QOL outcomes were analyzed using linear mixed-effects models adjusted for age, sex, and disease duration, with a patient-specific random intercept. All 47 patients contributed paired preoperative and postoperative observations. Eye-level associations between MRI and clinical changes were evaluated using generalized estimating equations with robust standard errors clustered by patient. Patient-level associations between MRI changes and GO-QOL changes were evaluated using multivariable linear regression after averaging MRI measurements across included eyes at each time point. Parsimonious and more-affected-eye sensitivity analyses were also performed. Further analytical details are provided in the Supplementary Statistical Methods. Representative residual diagnostic plots for the longitudinal mixed-effects models and primary patient-level MRI–GO–QOL association models are provided in Supplementary Figures 2, 3, respectively.

The Benjamini–Hochberg procedure was applied separately within prespecified hypothesis families. Both unadjusted *P*-values and FDR-adjusted *q*-values are reported, and q < 0.05 was considered statistically significant. Analyses were based on complete observations for the outcome and included covariates; no missing data were imputed. Hypothesis-family definitions, model-specific sample sizes, and model diagnostics are provided in the Supplementary Statistical Methods and Supplementary Table 3.

## 3 Results

### 3.1 Baseline and demographic characteristics at the patient level

All variables in Table 1 were analyzed at the patient level (*n* = 47). The cohort consisted of 27 females (57.4%) and 20 males (42.6%), with a mean age of 41.40 ± 13.97 years. The majority of patients were non-smokers (66.0%). Both eyes were included for 14 patients (29.8%). The median disease duration was 24.00 months (IQR, 7.00–60.00). Thyroid function, TG, and thyroid autoantibody levels are summarized in Table 1. The median GO-QOL score was 137.50 (IQR, 118.75–150.00), including an appearance score of 60.64 ± 9.66 and a function score of 81.25 (IQR, 50.00–87.50).

**TABLE 1:** Baseline demographic and clinical characteristics of patients (patient-level analysis).

Variables	Patients (*n* = 47)
Demographics	
Female, *n* (%)	27 (57.4)
Male, *n* (%)	20 (42.6)
Age (years)	41.40 ± 13.97
Smoking status	
- Non-smoker, *n* (%)	31 (66.0)
- Smoker, *n* (%)	16 (34.0)
Eye involvement	
One eye included, *n* (%)	33 (70.2)
Both eyes included, *n* (%)	14 (29.8)
Clinical characteristics	
Disease duration (months)	24.00 [7.00, 60.00]
TgAb (IU/mL)	22.05 [11.43, 75.99]
TPOAb (IU/mL)	26.32 [12.12, 245.20]
TRAb (IU/L)	4.29 [1.69, 8.30]
FT3 (pmol/L)	4.83 [4.25, 5.26]
FT4 (pmol/L)	16.32 [14.46, 18.16]
TSH (mIU/L)	1.41 [0.14, 2.93]
TG (ng/mL)	16.01 [0.68, 105.18]
GO-QOL	137.50 [118.75, 150.00]
Appearance	60.64 ± 9.66
Function	81.25 [50.00, 87.50]

TgAb, thyroglobulin antibody; TPOAb, thyroid peroxidase antibody; TRAb, thyrotropin receptor antibody; FT3, free triiodothyronine; FT4, free thyroxine; TSH, thyroid-stimulating hormone; TG, thyroglobulin; GO-QOL, Graves’ Ophthalmopathy Quality of Life Questionnaire. Values are presented as *n* (%), mean ± standard deviation, or median [interquartile range], as appropriate. Laboratory variables were summarized using available observations; variable-specific sample sizes are reported in Supplementary Table 3.

### 3.2 Baseline clinical and imaging characteristics at the eye level

As shown in Table 2 and Supplementary Table 4, the eye-level analysis included 61 eyes, comprising 34 TAO eyes and 27 DON eyes. After adjustment for age, sex, disease duration, and within-patient inter-eye correlation, DON eyes showed longer P100 latency and lower P100 amplitude, both of which remained significant after FDR correction. Although DON eyes had numerically higher logMAR BCVA, the adjusted between-group difference was not statistically significant. Lateral-wall-involving decompression was more frequent in DON eyes than in TAO eyes (85.2% vs. 61.8%; *P* < 0.001, *q* = 0.002). No other clinical characteristic differed significantly after FDR correction. FSADC, SIR, FA, VMI, and HMI showed nominal between-group differences, but no MRI parameter remained significant after FDR correction.

**TABLE 2:** Adjusted comparisons of clinical and imaging characteristics between TAO and DON eyes (eye-level analysis).

Variables	TAO eyes (*n* = 34)	DON eyes (*n* = 27)	*P*-value	*q*-value
Clinical features				
Strabismus, *n* (%)	9 (26.5)	16 (59.3)	0.132	0.132
Surgical type			<0.001	**0.002**
Lateral wall not included	13 (38.2)	4 (14.8)		
Lateral wall included	21 (61.8)	23 (85.2)		
NOSPECS	3.00 [2.75, 3.00]	3.00 [3.00, 4.00]	0.287	0.362
CAS	1.00 [1.00, 2.00]	2.00 [2.00, 4.00]	0.906	0.906
IOP (mmHg)	17.25 [15.00, 20.00]	19.00 [17.00, 25.00]	0.326	0.362
Degree (mm)	19.00 [18.00, 23.00]	20.00 [18.00, 22.00]	0.295	0.362
logMAR BCVA	0.00 [0.00, 0.02]	0.30 [0.10, 0.92]	0.055	0.183
P100				
Latency (ms)	105.80 [98.90, 110.50]	115.00 [93.45, 122.35]	0.004	**0.020**
Amplitude (μV)	7.56 [5.63, 11.69]	5.55 [2.59, 6.70]	0.003	**0.020**
VFI (%)	97.00 [93.00, 99.00]	78.50 [38.50, 93.25]	0.292	0.362
MD (dB)	−1.89 [−3.71, −0.29]	−8.07 [−17.37, −1.51]	0.107	0.214
PSD (dB)	2.12 [1.68, 3.66]	7.26 [2.48, 9.11]	0.087	0.214
Imaging parameters				
FA	0.51 [0.42, 0.56]	0.36 [0.31, 0.53]	0.029	0.149
ONDI	0.48 [0.45, 0.53]	0.45 [0.38, 0.52]	0.055	0.156
ONAI				
-C (coronal)	0.14 [0.12, 0.21]	0.16 [0.14, 0.21]	0.791	0.923
-A (axial)	0.48 [0.44, 0.53]	0.50 [0.40, 0.56]	0.491	0.896
-S (sagittal)	0.51 [0.49, 0.55]	0.51 [0.44, 0.57]	0.869	0.923
OAAI				
-V (vertical)	0.35 [0.30, 0.39]	0.41 [0.33, 0.47]	0.075	0.182
-H (horizontal)	0.31 [0.28, 0.36]	0.37 [0.31, 0.45]	0.860	0.923
SIR	0.98 [0.94, 1.04]	1.07 [0.96, 1.15]	0.028	0.149
FSADC (×10^−3^ mm^2^/s)	0.43 [0.38, 0.52]	0.56 [0.47, 0.69]	0.008	0.136
ONADC (×10^−3^ mm^2^/s)	1.12 [0.96, 1.25]	1.11 [0.99, 1.30]	0.966	0.966
SMADC (×10^−3^ mm^2^/s)	1.20 [1.05, 1.38]	1.34 [1.18, 1.53]	0.600	0.915
IMADC (×10^−3^ mm^2^/s)	1.10 [0.96, 1.39]	1.47 [1.11, 1.56]	0.377	0.801
LMADC (×10^−3^ mm^2^/s)	1.17 [1.08, 1.41]	1.24 [1.13, 1.54]	0.646	0.915
MMADC (×10^−3^ mm^2^/s)	1.17 [1.05, 1.46]	1.36 [1.10, 1.61]	0.825	0.923
MI				
VMI	0.38 [0.31, 0.45]	0.55 [0.44, 0.58]	0.035	0.149
HMI	0.36 [0.31, 0.46]	0.44 [0.39, 0.55]	0.046	0.156
COD (mm)	21.83 [20.25, 24.70]	22.46 [20.23, 25.46]	0.527	0.896

NOSPECS, NOSPECS classification; CAS, clinical activity score; IOP, intraocular pressure; Degree, proptosis degree; logMAR BCVA, logarithm of the minimum angle of resolution best-corrected visual acuity; VFI, visual field index; MD, mean deviation; PSD, pattern standard deviation; FA, fractional anisotropy; ONDI, optic nerve diameter index; ONAI, optic nerve area index (coronal/axial/sagittal); OAAI, orbital apex area index (vertical/horizontal); SIR, signal intensity ratio; FSADC, fat space apparent diffusion coefficient; ONADC, optic nerve apparent diffusion coefficient; SMADC/IMADC/LMADC/MMADC, superior/inferior/lateral/medial rectus muscle apparent diffusion coefficient; MI, muscle index; VMI, vertical muscle index; HMI, horizontal muscle index; COD, corneal protrusion distance. Values are presented as median [interquartile range] unless otherwise indicated. *P*-values were obtained from models adjusted for age, sex, and disease duration while accounting for within-patient inter-eye correlation. Continuous variables were analyzed using linear mixed-effects models, and categorical variables were analyzed using generalized estimating equations. Benjamini–Hochberg FDR correction was applied separately within the prespecified categorical clinical, continuous clinical/functional, and MRI families. *P*-values are unadjusted; *q*-values are FDR-adjusted. Bold values indicate q < 0.05.

### 3.3 Preoperative and postoperative comparisons

Longitudinal eye-level outcomes are summarized in Table 3. After multivariable adjustment, proptosis decreased by 2.35 mm (95% CI, −2.77 to −1.94; q < 0.001), and logMAR BCVA decreased by 0.088 (95% CI, −0.147 to −0.029; *q* = 0.016), indicating improved visual acuity. No significant changes were observed in IOP, electrophysiological measures, or visual-field indices after FDR correction.

**TABLE 3:** Longitudinal changes in clinical and imaging outcomes before and after surgery using multivariable mixed-effects models (eye-level analysis).

Variables	Preoperative adjusted mean	Postoperative adjusted mean	Mean difference	95% CI	*P*-value	*q*-value
Clinical features						
IOP (mmHg)	19.292	17.518	−1.774	−3.516 ∼−0.032	0.046	0.121
Degree (mm)	19.963	17.611	−2.352	−2.770 ∼−1.935	<0.001	**<0.001**
logMAR BCVA	0.304	0.216	−0.088	−0.147 ∼−0.029	0.004	**0.016**
P100						
Latency (ms)	109.508	111.043	1.535	−2.068 ∼ 5.138	0.398	0.455
Amplitude (μV)	6.684	4.970	−1.713	−3.728 ∼ 0.302	0.094	0.151
VFI (%)	76.615	82.636	6.021	−0.273 ∼ 12.315	0.060	0.121
MD (dB)	−6.457	−5.164	1.293	−0.897 ∼ 3.483	0.243	0.324
PSD (dB)	4.584	5.212	0.627	−1.209 ∼ 2.464	0.500	0.500
Imaging parameters						
FA	0.443	0.514	0.071	0.044 ∼ 0.098	<0.001	**<0.001**
ONDI	0.458	0.539	0.081	0.059 ∼ 0.103	<0.001	**<0.001**
ONAI						
-C (coronal)	0.160	0.199	0.038	0.027 ∼ 0.050	<0.001	**<0.001**
-A (axial)	0.497	0.605	0.107	0.091 ∼ 0.123	<0.001	**<0.001**
-S (sagittal)	0.511	0.599	0.088	0.073 ∼ 0.103	<0.001	**<0.001**
OAAI						
-V (vertical)	0.389	0.353	−0.036	−0.056 ∼−0.016	<0.001	**<0.001**
-H (horizontal)	0.361	0.319	−0.041	−0.062 ∼−0.021	<0.001	**<0.001**
SIR	1.019	1.025	0.005	−0.023 ∼ 0.033	0.700	0.700
FSADC (×10^−3^ mm^2^/s)	0.523	0.482	−0.041	−0.067 ∼−0.015	0.002	**0.003**
ONADC (×10^−3^ mm^2^/s)	1.134	1.271	0.137	0.076 ∼ 0.198	<0.001	**<0.001**
SMADC (×10^−3^ mm^2^/s)	1.295	1.246	−0.049	−0.100 ∼ 0.002	0.058	0.076
IMADC (×10^−3^ mm^2^/s)	1.232	1.192	−0.040	−0.105 ∼ 0.024	0.214	0.228
LMADC (×10^−3^ mm^2^/s)	1.260	1.306	0.046	−0.024 ∼ 0.116	0.191	0.217
MMADC (×10^−3^ mm^2^/s)	1.313	1.362	0.049	−0.018 ∼ 0.115	0.150	0.182
MI						
VMI	0.446	0.381	−0.065	−0.085 ∼−0.044	<0.001	**<0.001**
HMI	0.422	0.358	−0.064	−0.083 ∼−0.044	<0.001	**<0.001**
COD (mm)	22.474	19.375	−3.099	−3.577 ∼−2.621	<0.001	**<0.001**

IOP, intraocular pressure; Degree, proptosis degree; logMAR BCVA, logarithm of the minimum angle of resolution best-corrected visual acuity; VFI, visual field index; MD, mean deviation; PSD, pattern standard deviation; FA, fractional anisotropy; ONDI, optic nerve diameter index; ONAI, optic nerve area index (coronal/axial/sagittal); OAAI, orbital apex area index (vertical/horizontal); SIR, signal intensity ratio; FSADC, fat space apparent diffusion coefficient; ONADC, optic nerve apparent diffusion coefficient; SMADC/IMADC/LMADC/MMADC, superior/inferior/lateral/medial rectus muscle apparent diffusion coefficient; MI, muscle index; VMI, vertical muscle index; HMI, horizontal muscle index; COD, corneal protrusion distance. Values are adjusted estimated marginal means from the primary nested linear mixed-effects models fitted by restricted maximum likelihood using lmerTest::lmer(). Changes were calculated as postoperative minus preoperative values. The exact R formula was outcome ∼ time + age + sex + group + period + SurgType + strabismus + NOSPECS + (1 | patientid/eyeid), where group denoted disease subtype, period denoted disease duration, and SurgType denoted surgical approach. Degrees of freedom were estimated using the Satterthwaite method, and adjusted marginal means were obtained using emmeans with proportional weights. Benjamini–Hochberg FDR correction was applied separately across the 8 clinical and functional outcomes and the 17 MRI outcomes. *P*-values are unadjusted, and *q*-values are FDR-adjusted. Bold values indicate q < 0.05.

**FIGURE 4:**
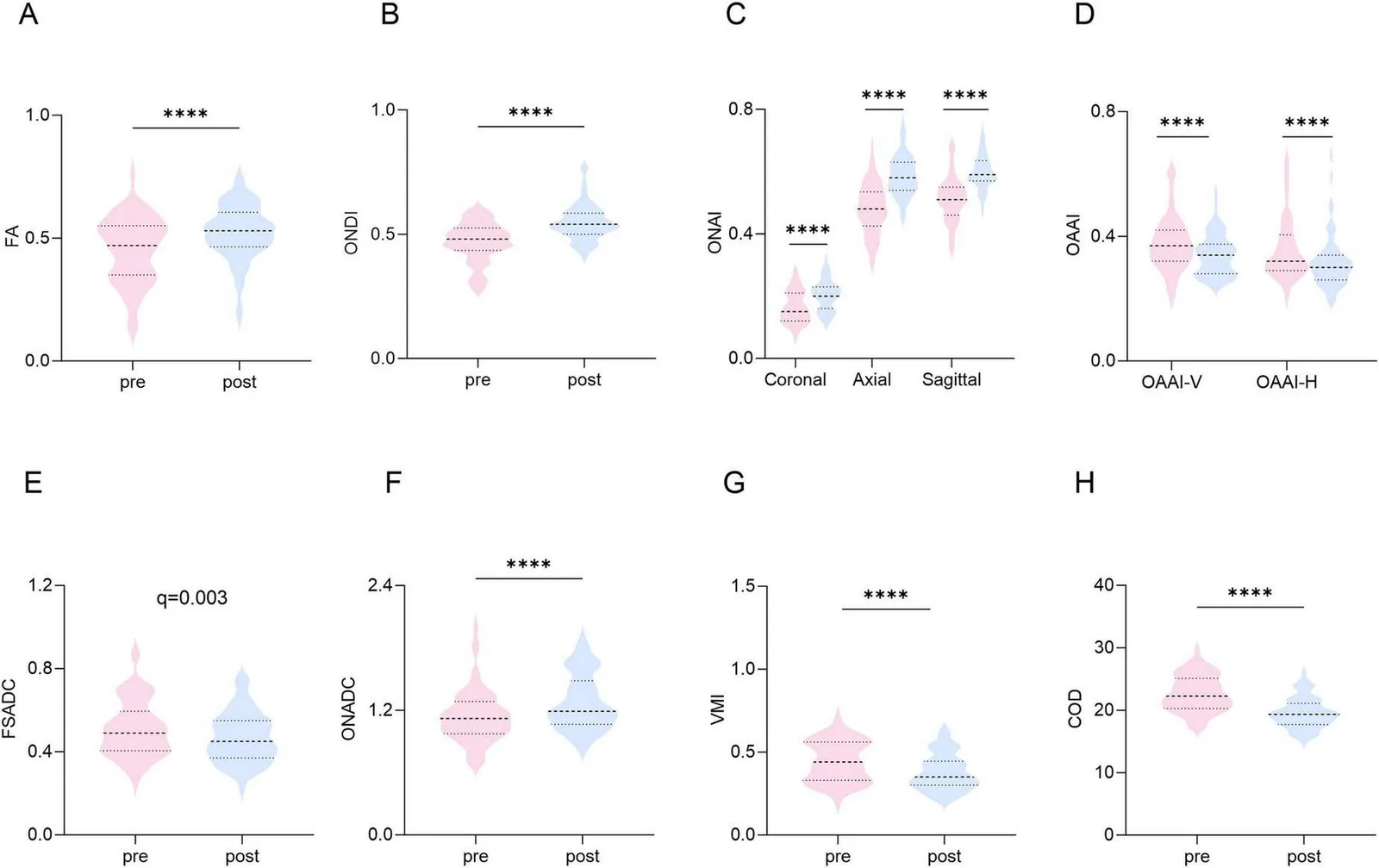
Changes in representative orbital MRI parameters before and after orbital decompression. **(A)** FA, **(B)** ONDI, **(C)** ONAI in the coronal, axial, and sagittal planes, **(D)** OAAI-V and OAAI-H, **(E)** FSADC, **(F)** ONADC, **(G)** VMI, and **(H)** COD. Violin plots show the distributions of values before surgery and at 6 months after surgery. Dashed lines indicate the median and interquartile range. Comparisons were performed using mixed-effects models, and significance annotations are based on FDR-adjusted *q*-values. ****, q < 0.001; the exact *q*-value is shown for FSADC (*q* = 0.003). FA, fractional anisotropy; ONDI, optic nerve diameter index; ONAI, optic nerve area index; OAAI-V/H, orbital apex area index (vertical/horizontal); FSADC, fat space apparent diffusion coefficient; ONADC, optic nerve apparent diffusion coefficient; VMI, vertical muscle index; COD, corneal protrusion distance.

Several MRI parameters changed significantly after surgery. FA, ONDI, ONAI-C, ONAI-A, ONAI-S, and ONADC increased, whereas OAAI-V, OAAI-H, COD, FSADC, VMI, and HMI decreased. SIR and rectus-muscle ADC parameters did not remain significant after FDR correction (Figure 4). All 25 primary nested mixed-effects models converged; eight were classified as singular because one or more random-effect variance components were estimated at or near zero. Detailed convergence and variance-component diagnostics are provided in Supplementary Table 5.

At the patient level, visual-function, appearance, and total GO-QOL scores increased by 9.71, 14.10, and 23.80 points, respectively, with all changes remaining significant after FDR correction (all q < 0.001; Supplementary Table 6).

In exploratory interaction analyses, logMAR BCVA changed by −0.011 in TAO eyes without DON and by −0.184 in DON eyes. The reported interaction coefficient was expressed as the TAO-minus-DON difference in change and was 0.173 (95% CI, 0.063–0.284; *P* = 0.003; *q* = 0.021). Because lower logMAR values indicate better visual acuity, the positive coefficient indicates a greater postoperative reduction and therefore greater visual-acuity improvement in DON eyes. No MRI time-by-disease-subtype interaction remained significant after FDR correction (Supplementary Table 7).

### 3.4 Associations between changes in clinical outcomes and MRI parameters

At the eye level, changes in proptosis were positively associated with changes in COD (*B* = 0.228, 95% CI, 0.100–0.355; *P* < 0.001; *q* = 0.008). No other eye-level association remained significant after FDR correction (Supplementary Table 8).

At the patient level, MRI values were averaged across included eyes at each time point. Greater reductions in OAAI-V and FSADC were associated with greater improvements in visual-function scores (OAAI-V: *B* = −55.554, 95% CI, −87.274 to −23.834, *q* = 0.017; FSADC: *B* = −33.037, 95% CI, −55.981 to −10.093, *q* = 0.0497). No MRI parameter was associated with changes in appearance scores after FDR correction. Greater reductions in OAAI-V and FSADC were also associated with greater improvements in total GO-QOL scores (OAAI-V: *B* = −100.228, 95% CI, −149.428 to −51.029, *q* = 0.003; FSADC: *B* = −59.102, 95% CI, −92.571 to −25.633, *q* = 0.008; Supplementary Table 9).

Parsimonious models reproduced these FDR-significant associations. In the more-affected-eye analysis, the directions of association were generally preserved but the estimates were attenuated; only the association between OAAI-V and total GO-QOL remained significant after FDR correction (Supplementary Table 10).

## 4 Discussion

This study identified multiple postoperative changes in orbital structural and diffusion-related MRI parameters following orbital decompression. FA, ONAI, ONDI, and ONADC increased, whereas OAAI, MI, COD, and FSADC decreased. These findings demonstrate measurable postoperative changes in orbital anatomy and tissue diffusion characteristics. At the eye level, greater reductions in COD were associated with greater reductions in proptosis after FDR correction, whereas most other MRI–clinical associations did not remain significant.

Among the imaging parameters, FA increased significantly after surgery. FA is a DTI-derived parameter reflecting the directional dependence of water diffusion and may be influenced by tissue organization, edema, inflammation, compression, and partial-volume effects (20, 21). Previous studies have reported inconsistent FA findings in TAO and DON, including lower FA in thyroid orbitopathy than in healthy controls, higher FA in TAO than in healthy controls, and lower FA in DON than in non-DON eyes (13, 22). In our study, the postoperative increase in FA may reflect changes in optic nerve organization, water content, or mechanical compression after decompression. However, because FA lacks pathological specificity, it should not be considered direct evidence of optic nerve fiber repair.

No significant FA time-by-disease-subtype interaction remained after FDR correction. Moreover, associations between FA changes and clinical outcomes were not consistently significant after multiple-comparison correction. Thus, although FA was sensitive to postoperative change, its value as an independent marker of functional recovery remains uncertain (23).

The patient-level analyses identified exploratory associations between reductions in OAAI-V and FSADC and improvement in the visual-function component of GO-QOL (24). OAAI-V showed the more consistent pattern across model specifications. Its associations remained significant in the parsimonious models and remained significant for total GO-QOL in the more-affected-eye sensitivity analysis. By contrast, the FSADC associations were retained in the primary and parsimonious models but were attenuated when MRI values from the functionally more affected eye were used. This finding suggests that the FSADC association may be more sensitive to how bilateral MRI information is summarized (25, 26).

These findings should not be interpreted as evidence that OAAI-V or FSADC independently predicts postoperative recovery. The analyses were exploratory, the patient-level sample size was modest, and MRI changes and GO-QOL changes were assessed during the same follow-up interval. The findings therefore support potential imaging correlates of patient-reported visual-function improvement rather than causal or predictive biomarkers.

The exploratory interaction analysis showed greater improvement in logMAR BCVA among DON eyes (27). This finding may partly reflect more severe preoperative visual impairment and greater potential for improvement. However, no MRI time-by-disease-subtype interaction remained significant after FDR correction. Therefore, the greater visual-acuity improvement in DON eyes was not accompanied by a statistically robust subtype-specific pattern in the evaluated MRI parameters.

This study has several limitations. First, the single-center retrospective design and modest patient-level sample size may limit generalizability and statistical power, particularly for exploratory MRI–GO-QOL association analyses. Second, the absence of a nonsurgical control group precludes separation of surgery-related changes from natural temporal variation. Third, the surgical approach was selected according to clinical indications and patient preference rather than random assignment, and residual confounding cannot be excluded. Fourth, MRI measurements were acquired using a single scanner and protocol, and external reproducibility remains to be established. Fifth, although the primary patient-level analyses used bilateral mean MRI values to correspond to patient-level GO-QOL outcomes, sensitivity analyses indicated that some findings, particularly those involving FSADC, depended on how bilateral MRI measurements were summarized. Finally, the 6-month follow-up period does not capture longer-term structural or functional changes.

## 5 Conclusion

Multimodal quantitative MRI detected postoperative structural and diffusion-related changes following orbital decompression in TAO. COD changes were associated with proptosis reduction, whereas reductions in OAAI-V and FSADC showed exploratory associations with patient-reported visual-function improvement, although the robustness of these associations differed across sensitivity analyses. These MRI parameters should therefore be considered complementary imaging measures rather than independent predictors of postoperative recovery.

## Data Availability

The original contributions presented in this study are included in this article/Supplementary material, further inquiries can be directed to the corresponding authors.

## Supplementary material

Supplementary Figure 1 Bland–Altman plots of intra-observer agreement for representative MRI parameters. **(A)** FA, **(B)** ONDI, **(C)** OAAI-V, and **(D)** FSADC. The solid blue line indicates the mean difference, the dashed red lines indicate the 95% limits of agreement, and the dotted black line indicates zero difference. Black points indicate observations outside the 95% limits of agreement. FA, fractional anisotropy; ONDI, optic nerve diameter index; OAAI-V, orbital apex area index in the vertical direction; FSADC, fat space apparent diffusion coefficient; LoA, limits of agreement.

Supplementary Figure 2 Residual diagnostics for representative longitudinal mixed-effects models. **(A,B)** Eye-level longitudinal FA model; **(C,D)** patient-level longitudinal Function GO-QOL model. **(A,C)** Show Q–Q plots of standardized residuals, and **(B,D)** show standardized residuals versus fitted values. The dashed horizontal line indicates zero residual. FA, fractional anisotropy; GO-QOL, Graves’ Ophthalmopathy Quality of Life questionnaire.

Supplementary Figure 3 Residual diagnostics for representative primary patient-level MRI–GO-QOL association models. **(A,B)** OAAI-V model for change in the Function GO-QOL score; **(C,D)** FSADC model for change in the Function GO-QOL score. **(A,C)** Show Q–Q plots of standardized residuals, and **(B,D)** show standardized residuals versus fitted values. The dashed horizontal line indicates zero residual. OAAI-V, orbital apex area index in the vertical direction; FSADC, fat space apparent diffusion coefficient; GO-QOL, Graves’ Ophthalmopathy Quality of Life questionnaire.
