# Supplementary material for: Changes in multimodal MRI parameters following orbital decompression surgery for thyroid-associated ophthalmopathy and their associations with clinical outcomes
Source: Front Med (Lausanne). 2026 Aug 17;13:1870430. doi: 10.3389/fmed.2026.1870430 (PMC13526559; doi:10.3389/fmed.2026.1870430)
Supplement: Supplementary file 1 [file Table_1.docx]

**Supplementary Table S1 MRI sequences and parameters**

| **Parameters** | **DTI** | **DWI** | **FS-T2WI** | **FS-T2WI** | **FS-T2WI** |
| --- | --- | --- | --- | --- | --- |
| Orientation | Axial | Coronal | Axial | Coronal | Sagittal |
| Echo time (ms) | 78 | 68 | 80 | 80 | 80 |
| Repetition time (ms) | 3528 | 2633 | 3000 | 3000 | 3000 |
| Flip angle (deg) | 90 | 90 | 90 | 90 | 90 |
| Field of view (mm) | 224×224×144 | 200×168×66 | 160×180×55 | 160×180×66 | 160×142×39 |
| Voxel size (mm³) | 2×2×2 | 1.6×1.81×4 | 0.7×0.7×2.5 | 0.6×0.75×2.5 | 0.55×0.55×2 |
| Matrix | 240×240 | 124×94 | 228×247 | 224×320 | 292×292 |
| Slice thickness (mm) | 2 | 4 | 2.5 | 2.5 | 2 |
| Slice number | 72 | 15 | 20 | 24 | 18 |
| b values (s/mm²) | 0/1000 | 0/800 | — | — | — |
| Directions | 96 | 3 | — | — | — |
| Acquisition time (s) | 351 | 158 | 84 | 90 | 120 |

MRI, magnetic resonance imaging; FS-T2WI, fat-suppressed T2-weighted imaging; DWI, diffusion-weighted imaging; DTI, diffusion tensor imaging.
