# Supplementary material for: Changes in multimodal MRI parameters following orbital decompression surgery for thyroid-associated ophthalmopathy and their associations with clinical outcomes
Source: Front Med (Lausanne). 2026 Aug 17;13:1870430. doi: 10.3389/fmed.2026.1870430 (PMC13526559; doi:10.3389/fmed.2026.1870430)
Supplement: Supplementary file 2 [file Table_10.docx]

**Supplementary Table S10 Sensitivity analyses of patient-level associations between MRI changes and GO-QOL changes**

**Panel A. Parsimonious models**

| **GO-QOL outcome** | **MRI parameter** | **B (95% CI)** | **Standardized β** | **P value** | **q value** |
| --- | --- | --- | --- | --- | --- |
| Function | OAAI-V | **−52.649 (−83.812 ~ −21.486)** | **−0.375** | **0.001** | **0.024** |
| Function | FSADC | **−32.784 (−55.289 ~ −10.278)** | **−0.292** | **0.005** | **0.045** |
| Appearance | OAAI-V | −43.024 (−76.665 ~ −9.382) | −0.486 | 0.013 | 0.114 |
| Appearance | FSADC | −30.023 (−52.579 ~ −7.467) | −0.424 | 0.010 | 0.114 |
| GO-QOL | OAAI-V | **−91.005 (−141.877 ~ −40.132)** | **−0.562** | **<0.001** | **0.008** |
| GO-QOL | FSADC | **−59.282 (−92.745 ~ −25.819)** | **−0.458** | **<0.001** | **0.008** |

**Panel B. More-affected-eye analysis**

| **GO-QOL outcome** | **MRI parameter** | **B (95% CI)** | **Standardized β** | **P value** | **q value** |
| --- | --- | --- | --- | --- | --- |
| Function | OAAI-V | −44.649 (−76.431 ~ −12.867) | −0.342 | 0.007 | 0.120 |
| Function | FSADC | −22.326 (−44.693 ~ 0.041) | −0.215 | 0.050 | 0.379 |
| Appearance | OAAI-V | −39.577 (−68.858 ~ −10.297) | −0.481 | 0.009 | 0.158 |
| Appearance | FSADC | −22.375 (−40.804 ~ −3.947) | −0.342 | 0.019 | 0.158 |
| GO-QOL | OAAI-V | **−85.261 (−134.895 ~ −35.626)** | **−0.566** | **0.001** | **0.021** |
| GO-QOL | FSADC | −44.178 (−76.714 ~ −11.643) | −0.369 | 0.009 | 0.077 |

GO-QOL, Graves' Ophthalmopathy Quality of Life Questionnaire; FSADC, fat space apparent diffusion coefficient; OAAI-V, orbital apex area index (vertical); DON, dysthyroid optic neuropathy; logMAR BCVA, logarithm of the minimum angle of resolution best-corrected visual acuity; VFI, visual field index.

Panel A presents parsimonious models adjusted for the corresponding baseline GO-QOL score and baseline MRI value. All models included 47 patients and had 43 residual degrees of freedom. Panel B used MRI changes from the functionally more affected eye and retained the same covariates as the primary models: the corresponding baseline GO-QOL score, baseline MRI value, age, and sex. The more affected eye was selected hierarchically as the DON eye; when both eyes had the same disease subtype, the eye with the higher baseline logMAR BCVA was selected, followed by the lower baseline VFI and then the lower eye ID in the event of further ties. All Panel B models included 47 patients and had 41 residual degrees of freedom. OAAI-V and FSADC are displayed across all three GO-QOL outcomes. Benjamini–Hochberg q values were calculated across all 17 MRI parameters separately within each GO-QOL outcome family and separately for each sensitivity analysis. P values are unadjusted, and q values are FDR-adjusted. Bold values indicate q < 0.05.
