# Supplementary material for: Changes in multimodal MRI parameters following orbital decompression surgery for thyroid-associated ophthalmopathy and their associations with clinical outcomes
Source: Front Med (Lausanne). 2026 Aug 17;13:1870430. doi: 10.3389/fmed.2026.1870430 (PMC13526559; doi:10.3389/fmed.2026.1870430)
Supplement: Supplementary file 3 [file Table_2.docx]

| **Parameters** | **Intra-observer ICC (95% CI)** | **Inter-observer ICC (95% CI)** |
| --- | --- | --- |
| FA | 0.992 (0.969–0.998) | 0.991 (0.965–0.998) |
| ONDI | 0.955 (0.804–0.989) | 0.984 (0.927–0.996) |
| ONAI-C | 0.974 (0.901–0.993) | 0.944 (0.800–0.986) |
| ONAI-A | 0.951 (0.827–0.987) | 0.958 (0.725–0.991) |
| ONAI-S | 0.975 (0.768–0.995) | 0.978 (0.867–0.995) |
| OAAI-V | 0.986 (0.948–0.996) | 0.990 (0.935–0.998) |
| OAAI-H | 0.974 (0.901–0.994) | 0.980 (0.925–0.995) |
| SIR | 0.987 (0.951–0.997) | 0.940 (0.780–0.985) |
| FSADC | 0.982 (0.931–0.995) | 0.983 (0.934–0.996) |
| ONADC | 0.973 (0.899–0.993) | 0.983 (0.934–0.996) |
| SMADC | 0.998 (0.993–1.000) | 0.994 (0.977–0.998) |
| IMADC | 0.999 (0.995–1.000) | 0.998 (0.993–1.000) |
| LMADC | 0.996 (0.985–0.999) | 0.972 (0.898–0.993) |
| MMADC | 0.997 (0.988–0.999) | 0.996 (0.984–0.999) |
| VMI | 0.982 (0.865–0.996) | 0.979 (0.919–0.995) |
| HMI | 0.989 (0.958–0.997) | 0.983 (0.935–0.996) |
| COD | 0.988 (0.932–0.997) | 0.979 (0.877–0.995) |

**Supplementary Table S2 Intra- and inter-observer reliability of 17 MRI parameters**

MRI, magnetic resonance imaging; ICC, intraclass correlation coefficient; CI, confidence interval; FA, fractional anisotropy; ONDI, optic nerve diameter index; ONAI, optic nerve area index (coronal/axial/sagittal); OAAI, orbital apex area index (vertical/horizontal); SIR, signal intensity ratio; FSADC, fat space apparent diffusion coefficient; ONADC, optic nerve apparent diffusion coefficient; SMADC/IMADC/LMADC/MMADC, superior/inferior/lateral/medial rectus muscle apparent diffusion coefficient; VMI, vertical muscle index; HMI, horizontal muscle index; COD, corneal protrusion distance.

All intra- and inter-observer ICC values were 0.940 or higher, indicating excellent measurement reliability. Intra-observer reliability was assessed using a two-way mixed-effects model with absolute agreement and single measurements. Inter-observer reliability was assessed using a two-way random-effects model with absolute agreement and single measurements. ICC values were interpreted as follows: <0.50, poor; 0.50-0.74, moderate; 0.75-0.90, good; and >0.90, excellent.
