# Supplementary material for: Changes in multimodal MRI parameters following orbital decompression surgery for thyroid-associated ophthalmopathy and their associations with clinical outcomes
Source: Front Med (Lausanne). 2026 Aug 17;13:1870430. doi: 10.3389/fmed.2026.1870430 (PMC13526559; doi:10.3389/fmed.2026.1870430)
Supplement: Supplementary file 4 [file Table_3.docx]

**Supplementary Table S3 Data structure, missing data, analytical sample sizes, and prespecified FDR hypothesis families**

**Panel A. Data structure**

| **Dataset** | **Unit of analysis** | **Unique patients, n** | **Unique eyes, n** | **Total records, n** |
| --- | --- | --- | --- | --- |
| Patient-level baseline data | Patient | 47 | — | 47 |
| Eye-level baseline data | Eye | 47 | 61 | 61 |
| Longitudinal eye-level data | Eye at each time point | 47 | 61 | 122 |
| Longitudinal GO-QOL data | Patient at each time point | 47 | — | 94 |
| Patient-level MRI–GO-QOL data | Patient | 47 | — | 47 |

**Panel B. Variables with missing data**

**Patient-level variables**

| **Variable** | **Total patients, n** | **Available, n** | **Missing, n** | **Missing, %** |
| --- | --- | --- | --- | --- |
| FT3 (pmol/L) | 47 | 45 | 2 | 4.3 |
| FT4 (pmol/L) | 47 | 45 | 2 | 4.3 |
| TSH (mIU/L) | 47 | 45 | 2 | 4.3 |
| TRAb (IU/L) | 47 | 46 | 1 | 2.1 |
| TG (ng/mL) | 47 | 46 | 1 | 2.1 |

**Longitudinal eye-level variables**

| **Variable** | **Total eye-time records, n** | **Available, n** | **Missing, n** | **Missing, %** |
| --- | --- | --- | --- | --- |
| P100 |  |  |  |  |
| Latency (ms) | 122 | 115 | 7 | 5.7 |
| Amplitude (µV) | 122 | 116 | 6 | 4.9 |
| VFI (%) | 122 | 115 | 7 | 5.7 |
| MD (dB) | 122 | 119 | 3 | 2.5 |
| PSD (dB) | 122 | 116 | 6 | 4.9 |

**Panel C. Complete observations included in multivariable analyses**

| **Analysis** | **Outcomes** | **Patients, n** | **Eyes, n** | **Records included, n** |
| --- | --- | --- | --- | --- |
| Baseline eye-level models |  |  |  |  |
|  | P100 latency | 44 | 56 | 56 |
|  | P100 amplitude | 45 | 58 | 58 |
|  | VFI | 45 | 58 | 58 |
|  | MD | 47 | 61 | 61 |
|  | PSD | 45 | 59 | 59 |
|  | Other eye-level outcomes | 47 | 61 | 61 |
| Longitudinal eye-level models |  |  |  |  |
|  | IOP, Degree, logMAR BCVA | 47 | 61 | 122 |
|  | P100 latency | 46 | 60 | 115 |
|  | P100 amplitude | 46 | 60 | 116 |
|  | VFI | 46 | 60 | 115 |
|  | MD | 47 | 61 | 119 |
|  | PSD | 47 | 61 | 116 |
|  | All MRI outcomes | 47 | 61 | 122 |
| Longitudinal GO-QOL models | Visual function, appearance, total GO-QOL | 47 | — | 94 |
| Eye-level MRI–clinical association models | All models | 47 | 61 | 61 |
| Primary patient-level MRI–GO-QOL models | All models | 47 | — | 47 |
| Parsimonious sensitivity models | All models | 47 | — | 47 |
| More-affected-eye sensitivity models | All models | 47 | — | 47 |

**Panel D. Prespecified hypothesis families for Benjamini–Hochberg FDR correction**

| **Analysis** | **Hypothesis family** | **Tests included** | **Number of tests** |  |
| --- | --- | --- | --- | --- |
| Baseline eye-level comparisons | Categorical clinical variables | Strabismus and surgical approach | 2 |  |
| Baseline eye-level comparisons | Continuous clinical and functional outcomes | NOSPECS, CAS, IOP, Degree, logMAR BCVA, P100 latency, P100 amplitude, VFI, MD, and PSD | 10 |  |
| Baseline eye-level comparisons | MRI parameters | FA, ONDI, ONAI-C, ONAI-A, ONAI-S, OAAI-V, OAAI-H, SIR, FSADC, ONADC, SMADC, IMADC, LMADC, MMADC, VMI, HMI, and COD | 17 |  |
| Longitudinal eye-level analyses | Clinical and functional outcomes | IOP, Degree, logMAR BCVA, P100 latency, P100 amplitude, VFI, MD, and PSD | 8 |  |
| Longitudinal eye-level analyses | MRI outcomes | The 17 MRI parameters listed above | 17 |  |
| Longitudinal patient-level analyses | GO-QOL outcomes | Visual function, appearance, and total GO-QOL scores | 3 |  |
| Time-by-disease-subtype interactions | Clinical and functional outcomes | The 8 clinical and functional outcomes listed above | 8 |  |
| Time-by-disease-subtype interactions | MRI outcomes | The 17 MRI parameters listed above | 17 |  |
| Eye-level MRI–clinical change associations | All MRI–clinical combinations | Two clinical outcomes × eight prespecified MRI parameters | 16 |  |
| Patient-level MRI–GO-QOL change associations | Visual-function GO-QOL | 17 MRI parameters | 17 |  |
| Patient-level MRI–GO-QOL change associations | Appearance GO-QOL | 17 MRI parameters | 17 |  |
| Patient-level MRI–GO-QOL change associations | Total GO-QOL | 17 MRI parameters | 17 |  |
| Parsimonious patient-level MRI–GO-QOL sensitivity analysis | Visual-function GO-QOL | 17 MRI parameters | 17 |  |
| Parsimonious patient-level MRI–GO-QOL sensitivity analysis | Appearance GO-QOL | 17 MRI parameters | 17 |  |
| Parsimonious patient-level MRI–GO-QOL sensitivity analysis | Total GO-QOL | 17 MRI parameters | 17 |  |
| More-affected-eye MRI–GO-QOL sensitivity analysis | Visual-function GO-QOL | 17 MRI parameters | 17 |  |
| More-affected-eye MRI–GO-QOL sensitivity analysis | Appearance GO-QOL | 17 MRI parameters | 17 |  |
| More-affected-eye MRI–GO-QOL sensitivity analysis | Total GO-QOL | 17 MRI parameters | 17 |  |

FT3, free triiodothyronine; FT4, free thyroxine; TSH, thyroid-stimulating hormone; TRAb, thyrotropin receptor antibody; TG, thyroglobulin; P100, P100 component of the visual evoked potential; VFI, visual field index; MD, mean deviation; PSD, pattern standard deviation; NOSPECS, NOSPECS classification; CAS, clinical activity score; IOP, intraocular pressure; logMAR BCVA, logarithm of the minimum angle of resolution best-corrected visual acuity; MRI, magnetic resonance imaging; FA, fractional anisotropy; ONDI, optic nerve diameter index; ONAI, optic nerve area index (coronal/axial/sagittal); OAAI, orbital apex area index (vertical/horizontal); SIR, signal intensity ratio; FSADC, fat space apparent diffusion coefficient; ONADC, optic nerve apparent diffusion coefficient; SMADC/IMADC/LMADC/MMADC, superior/inferior/lateral/medial rectus muscle apparent diffusion coefficient; VMI, vertical muscle index; HMI, horizontal muscle index; COD, corneal protrusion distance; GO-QOL, Graves’ Ophthalmopathy Quality of Life questionnaire; FDR, false discovery rate.

The study included 47 patients and 61 eyes, yielding 122 eye-time observations and 94 patient-time GO-QOL observations. Only variables with missing data are shown in Panel B. Model-specific sample sizes in Panel C represent observations with complete data for the outcome and all included covariates. No missing data were imputed. FDR correction was applied separately within each hypothesis family. Eye-level and patient-level analyses were corrected separately; patient-level MRI–GO-QOL associations were corrected separately within each GO-QOL outcome. The same outcome-specific correction was applied separately to the parsimonious and more-affected-eye sensitivity analyses. P values are unadjusted, whereas q values are Benjamini–Hochberg FDR-adjusted.
