# Supplementary material for: Changes in multimodal MRI parameters following orbital decompression surgery for thyroid-associated ophthalmopathy and their associations with clinical outcomes
Source: Front Med (Lausanne). 2026 Aug 17;13:1870430. doi: 10.3389/fmed.2026.1870430 (PMC13526559; doi:10.3389/fmed.2026.1870430)
Supplement: Supplementary file 5 [file Table_4.docx]

**Supplementary Table S4 Adjusted baseline clinical and MRI comparisons between TAO and DON eyes**

| **Variables** | **Adjusted mean TAO** | **Adjusted mean DON** | **Mean difference** | **95% CI** | **P value** | **FDR q value** |
| --- | --- | --- | --- | --- | --- | --- |
| **Outcomes with unadjusted P < 0.05** |  |  |  |  |  |  |
| P100 |  |  |  |  |  |  |
| Latency (ms) | 103.507 | 115.787 | -12.28 | -20.488 ~ -4.072 | 0.004 | **0.020** |
| Amplitude (µV) | 9.248 | 4.375 | 4.873 | 1.696 ~ 8.050 | 0.003 | **0.020** |
| FA | 0.488 | 0.395 | 0.093 | 0.010 ~ 0.176 | 0.029 | 0.149 |
| SIR | 0.983 | 1.06 | -0.076 | -0.144 ~ -0.009 | 0.028 | 0.149 |
| FSADC (×10⁻³ mm²/s) | 0.465 | 0.561 | -0.096 | -0.165 ~ -0.026 | 0.008 | 0.136 |
| VMI | 0.416 | 0.483 | -0.067 | -0.129 ~ -0.005 | 0.035 | 0.149 |
| HMI | 0.395 | 0.458 | -0.063 | -0.125 ~ -0.001 | 0.046 | 0.156 |
| **Other outcomes** | | |  |  |  |  |
| NOSPECS | 3.252 | 3.059 | 0.192 | -0.173 ~ 0.558 | 0.287 | 0.362 |
| CAS | 2.2 | 2.167 | 0.033 | -0.527 ~ 0.593 | 0.906 | 0.906 |
| logMAR BCVA | 0.159 | 0.413 | -0.254 | -0.514 ~ 0.005 | 0.055 | 0.183 |
| IOP (mmHg) | 19.096 | 21.205 | -2.109 | -6.385 ~ 2.168 | 0.326 | 0.362 |
| Degree (mm) | 19.703 | 20.48 | -0.777 | -2.255 ~ 0.702 | 0.295 | 0.362 |
| VFI (%) | 80.086 | 73.576 | 6.509 | -5.945 ~ 18.964 | 0.292 | 0.362 |
| MD (dB) | -4.509 | -7.558 | 3.048 | -0.677 ~ 6.774 | 0.107 | 0.214 |
| PSD (dB) | 3.98 | 5.872 | -1.892 | -4.068 ~ 0.285 | 0.087 | 0.214 |
| ONDI | 0.488 | 0.438 | 0.05 | -0.001 ~ 0.100 | 0.055 | 0.156 |
| ONAI |  |  |  |  |  |  |
| -C (coronal) | 0.166 | 0.162 | 0.004 | -0.029 ~ 0.038 | 0.791 | 0.923 |
| -A (axial) | 0.476 | 0.493 | -0.017 | -0.066 ~ 0.032 | 0.491 | 0.896 |
| -S (sagittal) | 0.509 | 0.505 | 0.004 | -0.043 ~ 0.050 | 0.869 | 0.923 |
| OAAI |  |  |  |  |  |  |
| -V(vertical) | 0.352 | 0.398 | -0.046 | -0.098 ~ 0.005 | 0.075 | 0.182 |
| -H(horizontal) | 0.352 | 0.357 | -0.005 | -0.061 ~ 0.051 | 0.860 | 0.923 |
| ONADC (×10⁻³ mm²/s) | 1.128 | 1.132 | -0.003 | -0.157 ~ 0.150 | 0.966 | 0.966 |
| SMADC (×10⁻³ mm²/s) | 1.254 | 1.291 | -0.037 | -0.177 ~ 0.103 | 0.600 | 0.915 |
| IMADC (×10⁻³ mm²/s) | 1.209 | 1.275 | -0.066 | -0.214 ~ 0.082 | 0.377 | 0.801 |
| LMADC (×10⁻³ mm²/s) | 1.25 | 1.283 | -0.033 | -0.175 ~ 0.109 | 0.646 | 0.915 |
| MMADC (×10⁻³ mm²/s) | 1.302 | 1.283 | 0.018 | -0.148 ~ 0.185 | 0.825 | 0.923 |
| COD (mm) | 22.653 | 23.126 | -0.473 | -1.976 ~ 1.029 | 0.527 | 0.896 |

NOSPECS, NOSPECS classification; CAS, clinical activity score; IOP, intraocular pressure; Degree, proptosis degree; logMAR BCVA, logarithm of the minimum angle of resolution best-corrected visual acuity; VFI, visual field index; MD, mean deviation; PSD, pattern standard deviation; FA, fractional anisotropy; ONDI, optic nerve diameter index; ONAI, optic nerve area index (coronal/axial/sagittal); OAAI, orbital apex area index (vertical/horizontal); SIR, signal intensity ratio; FSADC, fat space apparent diffusion coefficient; ONADC, optic nerve apparent diffusion coefficient; SMADC/IMADC/LMADC/MMADC, superior/inferior/lateral/medial rectus muscle apparent diffusion coefficient; MI, muscle index; VMI, vertical muscle index; HMI, horizontal muscle index; COD, corneal protrusion distance.

Adjusted means were estimated using linear mixed-effects models with disease subtype as the group effect and age, sex, and disease duration as covariates. Patient ID was included as a random intercept to account for correlation between fellow eyes. Mean differences were calculated as TAO eyes without DON minus DON eyes. Benjamini–Hochberg FDR correction was applied separately across the 10 clinical and functional outcomes and the 17 MRI parameters. P values are unadjusted, and q values are FDR-adjusted. Bold values indicate q < 0.05.
