# Supplementary material for: Changes in multimodal MRI parameters following orbital decompression surgery for thyroid-associated ophthalmopathy and their associations with clinical outcomes
Source: Front Med (Lausanne). 2026 Aug 17;13:1870430. doi: 10.3389/fmed.2026.1870430 (PMC13526559; doi:10.3389/fmed.2026.1870430)
Supplement: Supplementary file 6 [file Table_5.docx]

**Supplementary Table S5 Convergence and variance-component diagnostics for primary longitudinal nested mixed-effects models classified as singular**

| **Outcomes** | **Convergence status** | **Singular fit** | **Patient-level variance** | **Eye-within-patient variance** | **Boundary variance component** | **Time-effect P value** |
| --- | --- | --- | --- | --- | --- | --- |
| IOP (mmHg) | Converged | Yes | 14.2625 | 0 | Eye within patient | 0.046 |
| P100 latency (ms) | Converged | Yes | 105.5961 | <1×10^-6^ | Eye within patient | 0.398 |
| VFI (%) | Converged | Yes | 316.3259 | 0 | Eye within patient | 0.060 |
| MD (dB) | Converged | Yes | 3.8884 | 0 | Eye within patient | 0.243 |
| PSD (dB) | Converged | Yes | 0 | 0 | Patient and eye within patient | 0.500 |
| MMADC (×10⁻³ mm²/s) | Converged | Yes | 0.0262 | <1×10^-6^ | Eye within patient | 0.150 |
| VMI | Converged | Yes | <1×10^-6^ | 0.0042 | Patient | <0.001 |
| COD (mm) | Converged | Yes | 3.7374 | 0 | Eye within patient | <0.001 |

IOP, intraocular pressure; VFI, visual field index; MD, mean deviation; PSD, pattern standard deviation; MMADC, medial rectus muscle apparent diffusion coefficient; VMI, vertical muscle index; COD, corneal protrusion distance.

All 25 primary longitudinal outcomes were analyzed using nested linear mixed-effects models with random intercepts for patients and eyes nested within patients. All models completed optimization with convergence code 0 and without optimizer convergence failure. Eight models were classified as singular because one or more random-effect variance components were estimated at or near zero; the remaining 17 models were non-singular. Only singular models are presented. The fixed-effect estimates and FDR-adjusted conclusions are reported in Table 3.
