# Supplementary material for: Changes in multimodal MRI parameters following orbital decompression surgery for thyroid-associated ophthalmopathy and their associations with clinical outcomes
Source: Front Med (Lausanne). 2026 Aug 17;13:1870430. doi: 10.3389/fmed.2026.1870430 (PMC13526559; doi:10.3389/fmed.2026.1870430)
Supplement: Supplementary file 7 [file Table_6.docx]

**Supplementary Table S6 Adjusted longitudinal changes in GO-QOL after orbital decompression (patient-level analysis)**

| **Variables** | **Preoperative**  **adjusted mean** | **Postoperative adjusted mean** | **Mean difference** | **95% CI** | **P value** | **q value** |
| --- | --- | --- | --- | --- | --- | --- |
| GO-QOL | 131.78 | 155.59 | 23.80 | 20.20 ~ 27.41 | <0.001 | **<0.001** |
| Appearance | 60.64 | 74.73 | 14.10 | 12.13 ~ 16.06 | <0.001 | **<0.001** |
| Function | 71.14 | 80.85 | 9.71 | 6.58 ~ 12.83 | <0.001 | **<0.001** |

GO-QOL, Graves' Ophthalmopathy Quality of Life Questionnaire.

Values are adjusted estimated marginal means from patient-level mixed-effects models fitted by restricted maximum likelihood using lmerTest::lmer() with the formula outcome ~ time + age + sex + period + (1 | patientid). Changes were calculated as postoperative minus preoperative values. All 47 patients contributed complete preoperative and postoperative observations, yielding 94 patient-time observations. Benjamini–Hochberg FDR correction was applied across the three GO-QOL outcomes. P values are unadjusted, and q values are FDR-adjusted. Bold values indicate q < 0.05.
