# Supplementary material for: Changes in multimodal MRI parameters following orbital decompression surgery for thyroid-associated ophthalmopathy and their associations with clinical outcomes
Source: Front Med (Lausanne). 2026 Aug 17;13:1870430. doi: 10.3389/fmed.2026.1870430 (PMC13526559; doi:10.3389/fmed.2026.1870430)
Supplement: Supplementary file 8 [file Table_7.docx]

**Supplementary Table S7 Exploratory time-by-disease-subtype interaction analyses for longitudinal clinical and MRI outcomes**

| **Outcomes** | **TAO Δ (95% CI)** | **DON Δ (95% CI)** | **Difference in changes, β (95% CI)** | **P value** | **q value** |
| --- | --- | --- | --- | --- | --- |
| IOP (mmHg) | −2.015 (−4.363, 0.333) | −1.470 (−4.105, 1.165) | −0.544 (−4.074, 2.985) | 0.759 | 0.861 |
| Degree (mm) | −2.706 (−3.253, −2.159) | −1.907 (−2.522, −1.293) | −0.798 (−1.621, 0.024) | 0.057 | 0.144 |
| logMAR BCVA | **−0.011 (−0.085, 0.062)** | **−0.184 (−0.267, −0.102)** | **0.173 (0.063, 0.284)** | **0.003** | **0.021** |
| P100 latency (ms) | 4.796 (0.114, 9.477) | −2.690 (−7.999, 2.619) | 7.486 (0.428, 14.543) | 0.038 | 0.144 |
| P100 amplitude (µV) | −3.014 (−5.627, −0.401) | 0.091 (−2.982, 3.165) | −3.105 (−7.134, 0.923) | 0.128 | 0.205 |
| VFI (%) | 1.010 (−7.266, 9.286) | 12.462 (3.078, 21.845) | −11.452 (−23.965, 1.061) | 0.072 | 0.144 |
| MD (dB) | 0.247 (−2.695, 3.188) | 2.591 (−0.686, 5.867) | −2.344 (−6.747, 2.059) | 0.292 | 0.390 |
| PSD (dB) | 0.769 (−1.674, 3.212) | 0.438 (−2.383, 3.260) | 0.331 (−3.405, 4.067) | 0.861 | 0.861 |
| FA | 0.053 (0.017, 0.088) | 0.094 (0.055, 0.134) | −0.042 (−0.095, 0.011) | 0.121 | 0.395 |
| ONDI | 0.066 (0.037, 0.096) | 0.099 (0.067, 0.132) | −0.033 (−0.077, 0.011) | 0.139 | 0.395 |
| ONAI-C | 0.041 (0.025, 0.056) | 0.035 (0.018, 0.053) | 0.006 (−0.018, 0.029) | 0.625 | 0.759 |
| ONAI-A | 0.099 (0.078, 0.120) | 0.117 (0.094, 0.141) | −0.019 (−0.050, 0.013) | 0.242 | 0.412 |
| ONAI-S | 0.086 (0.065, 0.106) | 0.091 (0.068, 0.114) | −0.005 (−0.036, 0.025) | 0.737 | 0.829 |
| OAAI-V | −0.016 (−0.042, 0.009) | −0.061 (−0.089, −0.032) | 0.045 (0.006, 0.083) | 0.023 | 0.392 |
| OAAI-H | −0.033 (−0.060, −0.006) | −0.052 (−0.082, −0.022) | 0.019 (−0.022, 0.059) | 0.361 | 0.557 |
| SIR | 0.013 (−0.025, 0.051) | −0.004 (−0.046, 0.038) | 0.017 (−0.040, 0.074) | 0.549 | 0.718 |
| FSADC (×10⁻³ mm²/s) | −0.024 (−0.058, 0.010) | −0.063 (−0.101, −0.025) | 0.039 (−0.012, 0.091) | 0.133 | 0.395 |
| ONADC (×10⁻³ mm²/s) | 0.102 (0.020, 0.184) | 0.181 (0.089, 0.272) | −0.079 (−0.201, 0.044) | 0.204 | 0.412 |
| SMADC (×10⁻³ mm²/s) | −0.081 (−0.149, −0.013) | −0.009 (−0.086, 0.067) | −0.072 (−0.174, 0.030) | 0.164 | 0.399 |
| IMADC (×10⁻³ mm²/s) | −0.032 (−0.120, 0.055) | −0.051 (−0.149, 0.047) | 0.018 (−0.113, 0.149) | 0.780 | 0.829 |
| LMADC (×10⁻³ mm²/s) | 0.008 (−0.085, 0.101) | 0.094 (−0.011, 0.198) | −0.085 (−0.225, 0.054) | 0.226 | 0.412 |
| MMADC (×10⁻³ mm²/s) | 0.003 (−0.085, 0.091) | 0.106 (0.007, 0.205) | −0.103 (−0.236, 0.030) | 0.126 | 0.395 |
| VMI | −0.048 (−0.075, −0.021) | −0.086 (−0.116, −0.055) | 0.038 (−0.003, 0.079) | 0.071 | 0.395 |
| HMI | −0.057 (−0.084, −0.031) | −0.072 (−0.102, −0.043) | 0.015 (−0.024, 0.054) | 0.453 | 0.641 |
| COD (mm) | −3.144 (−3.789, −2.499) | −3.043 (−3.766, −2.319) | −0.101 (−1.071, 0.868) | 0.836 | 0.836 |

IOP, intraocular pressure; Degree, proptosis degree; logMAR BCVA, logarithm of the minimum angle of resolution best-corrected visual acuity; VFI, visual field index; MD, mean deviation; PSD, pattern standard deviation; FA, fractional anisotropy; ONDI, optic nerve diameter index; ONAI, optic nerve area index (coronal/axial/sagittal); OAAI, orbital apex area index (vertical/horizontal); SIR, signal intensity ratio; FSADC, fat space apparent diffusion coefficient; ONADC, optic nerve apparent diffusion coefficient; SMADC/IMADC/LMADC/MMADC, superior/inferior/lateral/medial rectus muscle apparent diffusion coefficient; VMI, vertical muscle index; HMI, horizontal muscle index; COD, corneal protrusion distance.

Values are adjusted changes from baseline to 6 months after surgery and are presented as estimated marginal differences with 95% CIs from nested linear mixed-effects models. Changes were calculated as postoperative minus preoperative values. The exact R formula was outcome ~ time * group + age + sex + period + SurgType + strabismus + NOSPECS + (1 | patientid/eyeid), where group denoted disease subtype, period denoted disease duration, and SurgType denoted surgical approach. The interaction coefficient is reported as the TAO-minus-DON difference in change (ΔTAO − ΔDON). For logMAR BCVA, lower values indicate better visual acuity; therefore, a positive interaction coefficient indicates a larger postoperative reduction and greater visual-acuity improvement in DON eyes. Benjamini–Hochberg FDR correction was applied separately across the 8 clinical and functional interactions and the 17 MRI interactions. All 25 models completed optimization with convergence code 0; eight were classified as singular because one or more variance components were estimated at or near zero. P values are unadjusted, and q values are FDR-adjusted. Bold values indicate q < 0.05.
