# Supplementary material for: Changes in multimodal MRI parameters following orbital decompression surgery for thyroid-associated ophthalmopathy and their associations with clinical outcomes
Source: Front Med (Lausanne). 2026 Aug 17;13:1870430. doi: 10.3389/fmed.2026.1870430 (PMC13526559; doi:10.3389/fmed.2026.1870430)
Supplement: Supplementary file 9 [file Table_8.docx]

**Supplementary Table S8 Eye-level associations between changes in clinical outcomes and MRI parameters**

| **Clinical outcomes** | **MRI parameters** | **B (95% CI)** | **P value** | **q value** |
| --- | --- | --- | --- | --- |
| Change in Degree |  |  |  |  |
|  | FA | 1.237 (−2.599 ~ 5.074) | 0.527 | 0.882 |
|  | ONDI | 1.267 (−2.904 ~ 5.438) | 0.552 | 0.882 |
|  | ONAI-A | −1.291 (−8.160 ~ 5.578) | 0.713 | 0.882 |
|  | OAAI-H | −5.449 (−9.732 ~ −1.166) | 0.013 | 0.101 |
|  | FSADC | 0.116 (−4.568 ~ 4.800) | 0.961 | 0.961 |
|  | ONADC | −0.241 (−2.329 ~ 1.848) | 0.821 | 0.897 |
|  | VMI | 0.885 (−3.892 ~ 5.662) | 0.716 | 0.882 |
|  | **COD** | **0.228 (0.100 ~ 0.355)** | **<0.001** | **0.008** |
| Change in logMAR BCVA |  |  |  |  |
|  | FA | −0.218 (−0.758 ~ 0.323) | 0.430 | 0.882 |
|  | ONDI | 0.453 (−0.302 ~ 1.207) | 0.240 | 0.767 |
|  | ONAI-A | 0.466 (−0.082 ~ 1.015) | 0.096 | 0.382 |
|  | OAAI-H | −0.190 (−1.108 ~ 0.729) | 0.685 | 0.882 |
|  | FSADC | −0.123 (−0.508 ~ 0.263) | 0.532 | 0.882 |
|  | ONADC | 0.021 (−0.185 ~ 0.227) | 0.841 | 0.897 |
|  | VMI | −0.142 (−0.787 ~ 0.503) | 0.667 | 0.882 |
|  | COD | 0.029 (0.004 ~ 0.054) | 0.022 | 0.117 |

logMAR BCVA, logarithm of the minimum angle of resolution best-corrected visual acuity; Degree, proptosis degree; FA, fractional anisotropy; ONDI, optic nerve diameter index; ONAI-A, optic nerve area index measured on axial images; OAAI-H, orbital apex area index measured in the horizontal plane; FSADC, fat space apparent diffusion coefficient; ONADC, optic nerve apparent diffusion coefficient; VMI, vertical muscle index; COD, corneal protrusion distance; CI, confidence interval; FDR, false discovery rate.

Changes were calculated as postoperative minus preoperative values. Each MRI parameter was evaluated in a separate generalized estimating equation model with a Gaussian distribution, identity link, independent working correlation structure, and robust standard errors clustered by patient. Models were adjusted for the corresponding baseline clinical outcome, corresponding baseline MRI parameter, age, sex, disease subtype, disease duration, surgical approach, strabismus status, and preoperative NOSPECS classification. All models included 61 eyes from 47 patients. Benjamini–Hochberg FDR correction was applied across the 16 eye-level associations. P values are unadjusted, and q values are FDR-adjusted. Bold values indicate q < 0.05.
