# Supplementary material for: Changes in multimodal MRI parameters following orbital decompression surgery for thyroid-associated ophthalmopathy and their associations with clinical outcomes
Source: Front Med (Lausanne). 2026 Aug 17;13:1870430. doi: 10.3389/fmed.2026.1870430 (PMC13526559; doi:10.3389/fmed.2026.1870430)
Supplement: Supplementary file 10 [file Table_9.docx]

**Supplementary Table S9 Patient-level associations between MRI changes and GO-QOL changes**

| **Variables** | **B (95% CI)** | **Standardized β** | **P value** | **q value** |
| --- | --- | --- | --- | --- |
| **Function** |  |  |  |  |
| FA | 23.209 (−0.619 ~ 47.037) | 0.227 | 0.056 | 0.238 |
| ONDI | 11.388 (−25.005 ~ 47.781) | 0.087 | 0.531 | 0.906 |
| ONAI-C | 31.371 (−23.793 ~ 86.534) | 0.120 | 0.258 | 0.730 |
| ONAI-A | 9.423 (−37.939 ~ 56.786) | 0.049 | 0.690 | 0.906 |
| ONAI-S | 10.977 (−40.641 ~ 62.596) | 0.061 | 0.670 | 0.906 |
| OAAI-V | **−55.554 (−87.274 ~ −23.834)** | **−0.395** | **0.001** | **0.017** |
| OAAI-H | −39.405 (−76.996 ~ −1.815) | −0.245 | 0.040 | 0.229 |
| SIR | −3.218 (−30.673 ~ 24.238) | −0.030 | 0.814 | 0.934 |
| FSADC (×10⁻³ mm²/s) | **−33.037 (−55.981 ~ −10.093)** | **−0.294** | **0.006** | **0.0497** |
| ONADC (×10⁻³ mm²/s) | −0.427 (−10.841 ~ 9.986) | −0.009 | 0.934 | 0.934 |
| SMADC (×10⁻³ mm²/s) | −1.022 (−14.077 ~ 12.033) | −0.018 | 0.875 | 0.934 |
| IMADC (×10⁻³ mm²/s) | −3.210 (−14.752 ~ 8.331) | −0.073 | 0.577 | 0.906 |
| LMADC (×10⁻³ mm²/s) | 3.618 (−6.475 ~ 13.712) | 0.087 | 0.473 | 0.906 |
| MMADC (×10⁻³ mm²/s) | 5.847 (−6.044 ~ 17.739) | 0.138 | 0.327 | 0.793 |
| VMI | −26.770 (−59.558 ~ 6.018) | −0.183 | 0.107 | 0.363 |
| HMI | 1.475 (−34.046 ~ 36.996) | 0.010 | 0.933 | 0.934 |
| COD (mm) | 0.309 (−1.259 ~ 1.878) | 0.049 | 0.693 | 0.906 |
| **Appearance** |  |  |  |  |
| FA | 17.506 (−2.066 ~ 37.077) | 0.272 | 0.078 | 0.353 |
| ONDI | 4.492 (−25.451 ~ 34.434) | 0.055 | 0.763 | 0.865 |
| ONAI-C | −4.026 (−52.669 ~ 44.616) | −0.024 | 0.868 | 0.922 |
| ONAI-A | 28.624 (−11.399 ~ 68.647) | 0.237 | 0.156 | 0.443 |
| ONAI-S | −11.294 (−53.821 ~ 31.232) | −0.099 | 0.595 | 0.752 |
| OAAI-V | −43.361 (−73.214 ~ −13.507) | −0.490 | 0.005 | 0.093 |
| OAAI-H | −26.634 (−56.901 ~ 3.634) | −0.263 | 0.083 | 0.353 |
| SIR | −5.377 (−27.070 ~ 16.316) | −0.079 | 0.619 | 0.752 |
| FSADC (×10⁻³ mm²/s) | −26.428 (−46.618 ~ −6.238) | −0.374 | 0.012 | 0.098 |
| ONADC (×10⁻³ mm²/s) | −2.557 (−10.830 ~ 5.717) | −0.090 | 0.536 | 0.752 |
| SMADC (×10⁻³ mm²/s) | −3.261 (−14.121 ~ 7.599) | −0.090 | 0.548 | 0.752 |
| IMADC (×10⁻³ mm²/s) | −3.000 (−13.236 ~ 7.236) | −0.108 | 0.557 | 0.752 |
| LMADC (×10⁻³ mm²/s) | −3.139 (−11.067 ~ 4.790) | −0.120 | 0.429 | 0.752 |
| MMADC (×10⁻³ mm²/s) | −6.981 (−16.426 ~ 2.463) | −0.263 | 0.143 | 0.443 |
| VMI | −0.967 (−32.014 ~ 30.080) | −0.011 | 0.950 | 0.950 |
| HMI | −12.937 (−40.305 ~ 14.432) | −0.144 | 0.345 | 0.733 |
| COD (mm) | −0.657 (−1.901 ~ 0.588) | −0.167 | 0.293 | 0.711 |
| **GO-QOL** |  |  |  |  |
| FA | 38.725 (2.170 ~ 75.279) | 0.329 | 0.038 | 0.163 |
| ONDI | 15.910 (−40.587 ~ 72.407) | 0.106 | 0.573 | 0.941 |
| ONAI-C | 28.709 (−59.540 ~ 116.958) | 0.095 | 0.515 | 0.941 |
| ONAI-A | 31.757 (−41.390 ~ 104.905) | 0.144 | 0.386 | 0.941 |
| ONAI-S | −3.762 (−85.544 ~ 78.019) | −0.018 | 0.926 | 0.941 |
| OAAI-V | **−100.228 (−149.428 ~ −51.029)** | **−0.619** | **<0.001** | **0.003** |
| OAAI-H | −62.275 (−120.055 ~ −4.494) | −0.336 | 0.035 | 0.163 |
| SIR | −7.923 (−49.430 ~ 33.584) | −0.063 | 0.702 | 0.941 |
| FSADC (×10⁻³ mm²/s) | **−59.102 (−92.571 ~ −25.633)** | **−0.457** | **0.001** | **0.008** |
| ONADC (×10⁻³ mm²/s) | −2.492 (−18.670 ~ 13.686) | −0.048 | 0.757 | 0.941 |
| SMADC (×10⁻³ mm²/s) | −2.138 (−22.395 ~ 18.120) | −0.032 | 0.832 | 0.941 |
| IMADC (×10⁻³ mm²/s) | −6.442 (−25.144 ~ 12.259) | −0.126 | 0.491 | 0.941 |
| LMADC (×10⁻³ mm²/s) | 2.004 (−13.548 ~ 17.556) | 0.042 | 0.796 | 0.941 |
| MMADC (×10⁻³ mm²/s) | 0.687 (−17.687 ~ 19.060) | 0.014 | 0.94 | 0.941 |
| VMI | −32.513 (−85.594 ~ 20.569) | −0.193 | 0.223 | 0.759 |
| HMI | −7.596 (−63.443 ~ 48.251) | −0.046 | 0.785 | 0.941 |
| COD (mm) | −0.198 (−2.593 ~ 2.196) | −0.028 | 0.868 | 0.941 |

GO-QOL, Graves' Ophthalmopathy Quality of Life Questionnaire; FA, fractional anisotropy; ONDI, optic nerve diameter index; ONAI, optic nerve area index (coronal/axial/sagittal); OAAI, orbital apex area index (vertical/horizontal); SIR, signal intensity ratio; FSADC, fat space apparent diffusion coefficient; ONADC, optic nerve apparent diffusion coefficient; SMADC/IMADC/LMADC/MMADC, superior/inferior/lateral/medial rectus muscle apparent diffusion coefficient; VMI, vertical muscle index; HMI, horizontal muscle index; COD, corneal protrusion distance.

MRI values were averaged across included eyes at each time point, and changes were calculated as postoperative minus preoperative values. Separate multivariable linear regression models were fitted for each MRI parameter and GO-QOL outcome, with adjustment for the corresponding baseline GO-QOL score, baseline MRI value, age, and sex. All models included 47 patients and had 41 residual degrees of freedom. Standardized β values were calculated after standardization of continuous variables. The Benjamini–Hochberg procedure was applied separately within the visual-function, appearance, and total GO-QOL outcome families, with 17 MRI parameters included in each family. P values are unadjusted, and q values are FDR-adjusted. Bold values indicate q < 0.05.
